# Adeno-associated virus: from defective virus to effective vector

**DOI:** 10.1186/1743-422X-2-43

**Published:** 2005-05-06

**Authors:** Manuel AFV Gonçalves

**Affiliations:** 1 Gene Therapy Section, Department of Molecular Cell Biology, Leiden University Medical Center, Wassenaarseweg 72, 2333 AL Leiden, the Netherlands

## Abstract

The initial discovery of adeno-associated virus (AAV) mixed with adenovirus particles was not a fortuitous one but rather an expression of AAV biology. Indeed, as it came to be known, in addition to the unavoidable host cell, AAV typically needs a so-called helper virus such as adenovirus to replicate. Since the AAV life cycle revolves around another unrelated virus it was dubbed a satellite virus. However, the structural simplicity plus the defective and non-pathogenic character of this satellite virus caused recombinant forms to acquire centre-stage prominence in the current constellation of vectors for human gene therapy. In the present review, issues related to the development of recombinant AAV (rAAV) vectors, from the general principle to production methods, tropism modifications and other emerging technologies are discussed. In addition, the accumulating knowledge regarding the mechanisms of rAAV genome transduction and persistence is reviewed. The topics on rAAV vectorology are supplemented with information on the parental virus biology with an emphasis on aspects that directly impact on vector design and performance such as genome replication, genetic structure, and host cell entry.

## Adeno-associated virus biology

### Genome structure, DNA replication and virus assembly

**Figure 1:**
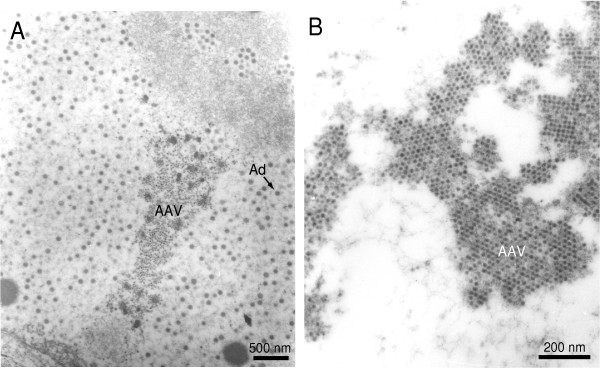
Transmission electron microscopy of AAV2 and Ad5 particles in human cells. (A) AAV2 and Ad5 particles in the nucleus of a HeLa cell at 48 hours after co-infection. Magnification: × 15,000. (B) AAV2 virions in a HeLa cell at 48 hours after co-infection with Ad5. Magnification: × 40,000.

**Figure 2:**
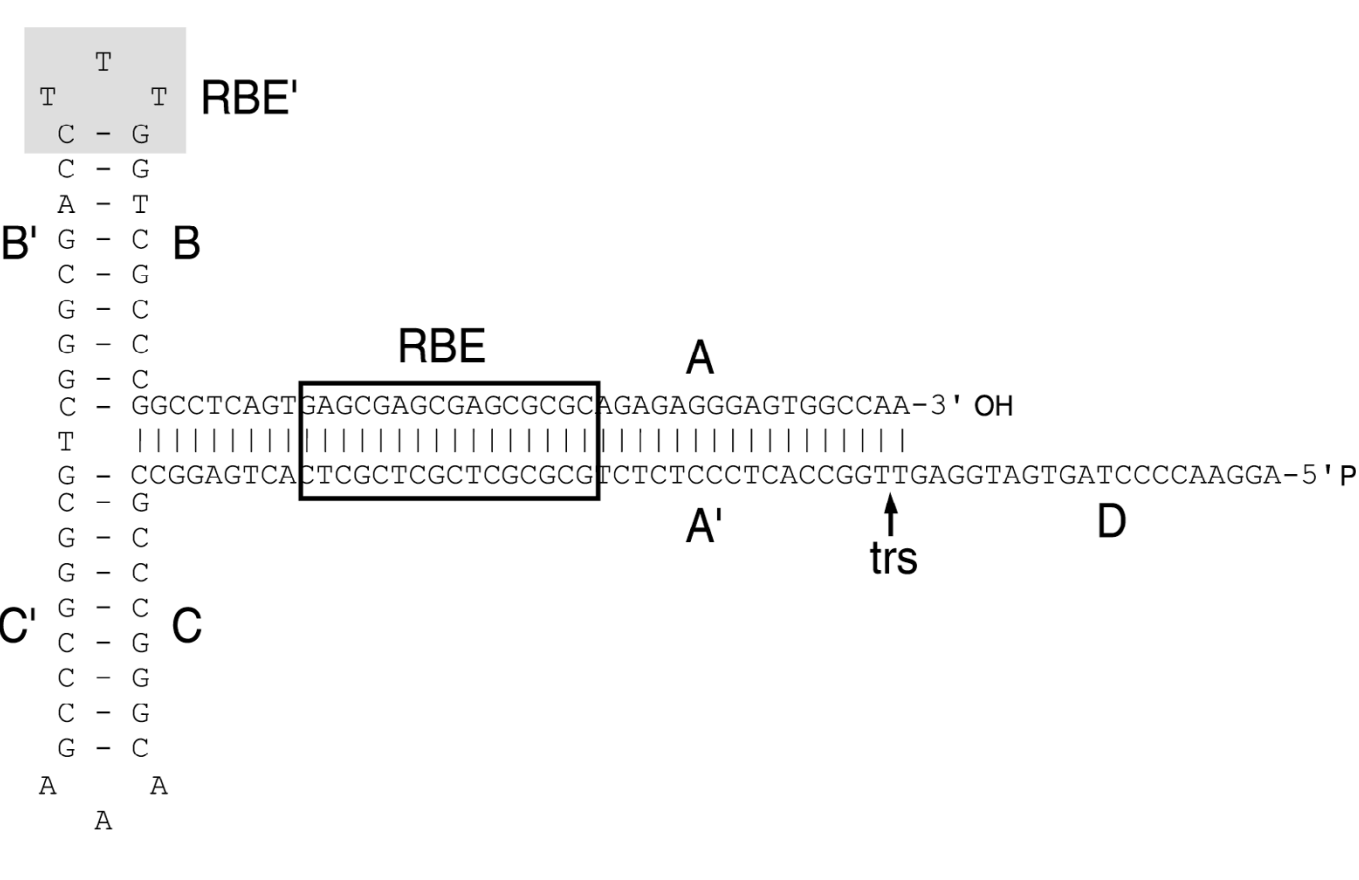
Secondary structure of the AAV2 ITR. The AAV2 ITR serves as origin of replication and is composed of two arm palindromes (B-B' and C-C') embedded in a larger stem palindrome (A-A'). The ITR can acquire two configurations (flip and flop). The flip (depicted) and flop configurations have the B-B' and the C-C' palindrome closest to the 3' end, respectively. The D sequence is present only once at each end of the genome thus remaining single-stranded. The boxed motif corresponds to the Rep-binding element (RBE) [119] where the AAV Rep78 and Rep68 proteins bind. The RBE consists of a tetranucleotide repeat with the consensus sequence 5'-GNGC-3'. The ATP-dependent DNA helicase activities of Rep78 and Rep68 remodel the A-A' region generating a stem-loop that locates at the summit the terminal resolution site (trs) in a single-stranded form [120,121]. In this configuration, the strand- and site-specific endonuclease catalytic domain of Rep78 and Rep68 introduces a nick at the trs. The shaded nucleotides at the apex of the T-shaped structure correspond to an additional RBE (RBE') [121] that stabilizes the association between the two largest Rep proteins and the ITR.

**Figure 3:**
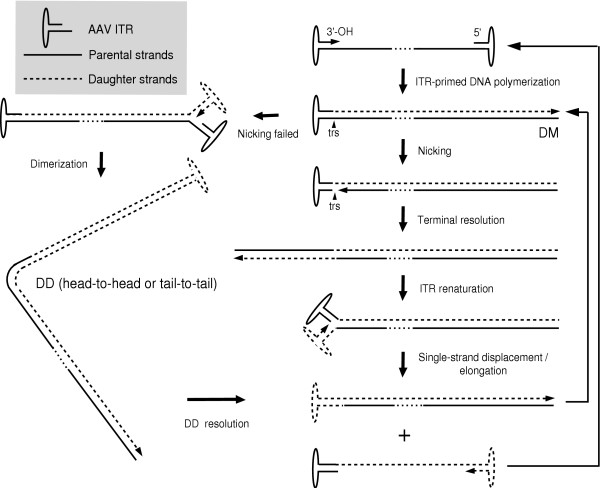
Schematic representation of the AAV DNA replication model. AAV DNA replication is thought to involve a self-priming single-strand displacement mechanism that is initiated by DNA polymerisation at the 3' hairpin primer of input single-stranded genomes. This leads to the formation of linear unit-length double-stranded molecules (duplex monomers, DMs) with one covalently closed end. These structures are resolved at the terminal resolution site (trs) by site-specific nicking of the parental strand opposite the original 3' end position (i.e., at nucleotide 125). The newly generated free 3' hydroxyl groups provide a substrate for DNA polymerases that unwind and copy the inverted terminal repeat (ITR). Finally, the palindromic linear duplex termini can renaturate into terminal hairpins putting the 3' hydroxyl groups in position for single-strand displacement synthesis. Next, single-stranded genomes and new DM replicative forms are made. When nicking does not occur, elongation proceeds through the covalently closed hairpin structure generating linear double-length double-stranded molecules (duplex dimers, DDs) with either a head-to-head or a tail-to-tail configuration. The DD replicative intermediates can be resolved to DMs through the AAV ITR sequences located at the axis of symmetry.

The human adeno-associated virus (AAV) was discovered in 1965 as a contaminant of adenovirus (Ad) preparations [1]. AAV is one of the smallest viruses with a non-enveloped icosahedral capsid of approximately 22 nm (Fig. 1), the crystal structure of which has been recently determined to a 3-angstrom resolution [2]. Because a co-infecting helper virus is usually required for a productive infection to occur, AAV serotypes are ascribed to a separate genus in the *Parvoviridae *family designated *Dependovirus*. Despite the high seroprevalence of AAV in the human population (approximately 80% of humans are seropositive for AAV2) the virus has not been linked to any human illness. The AAV has a linear single-stranded DNA genome of approximately 4.7-kilobases (kb). The AAV2 DNA termini consist of a 145 nucleotide-long inverted terminal repeat (ITR) that, due to the multipalindromic nature of its terminal 125 bases, can fold on itself via complementary Watson-Crick base pairing and form a characteristic T-shaped hairpin structure (Fig. 2) [3]. According to the AAV DNA replication model [4] this secondary structure provides a free 3' hydroxyl group for the initiation of viral DNA replication via a self-priming strand-displacement mechanism involving leading-strand synthesis and double-stranded replicative intermediates (Fig. 3). The virus does not encode a polymerase relying instead on cellular polymerase activities to replicate its DNA [5]. The ITRs flank the two viral genes *rep *(replication) and *cap *(capsid) encoding nonstructural and structural proteins, respectively. The *rep *gene, through the use of two promoters located at map positions 5 (p5) and 19 (p19), and an internal splice donor and acceptor site, encode four regulatory proteins that are dubbed Rep78, Rep68, Rep52 and Rep40 on basis of their apparent molecular weights. The Rep78 and Rep68 proteins participate in the AAV DNA replication process via their interaction with Rep-binding element (RBE) and terminal resolution site (trs) sequences located within the ITRs (Fig. 2). In addition, in response to environmental cues such as presence or absence of a helper virus these proteins either positively or negatively regulate AAV gene expression, respectively [6]. The Rep52 and Rep40 proteins are involved in the generation and accumulation of single-stranded viral genomes from double-stranded replicative intermediates [7]. The resulting single-stranded genomes with plus and minus polarities are packaged with equal efficiency [8]. The economy displayed by AAV is staggering and derives not only from its overlapping genetic organization but also from the integration of various biochemical activities in each of its few gene products. For instance, Rep78 and Rep68 are site-specific DNA binding proteins, as well as strand- and site-specific endonucleases [9]. They also exhibit helicase and ATPase activities [10], which are shared by Rep52 [11] and by Rep40 [12].

The *cap *gene is transcribed from a single promoter at map position 40 (p40). Alternative splicing at two acceptor sites originates two transcripts. The larger transcript encodes virion protein 1 (VP1), the biggest capsid protein subunit. The shorter mRNA possesses a noncanonical start codon (ACG), which is utilized to generate VP2, and a downstream conventional initiation codon (AUG) directing the synthesis of VP3. The VP1, VP2 and VP3 proteins differ from each other at their N terminus and have apparent molecular masses of 87, 72 and 62 kDa, respectively. Together they assemble into a near-spherical protein shell of 60 subunits with T = 1 icosahedral symmetry. At the 12 fivefold axes of symmetry lay narrow pores lately shown to be instrumental for virus infectivity and for genome packaging [13]. The molar ratio between VP1, VP2 and VP3 in AAV particles is 1:1:10. This stoichiometry is thought to reflect the relative abundance of the two *cap *gene transcripts and the relative efficiency of translation initiation at the three start codons for the structural proteins. A conserved phospholipase A_2 _(PLA2) motif, initially identified within the unique N-terminal region of the parvoviral VP1 proteins [14], was also reported to have a biological significance in AAV2 infection [15]. Specifically, although dispensable for capsid assembly, DNA packaging, and virion internalisation, the VP1-embedded PLA2 activity seems to play a key role at some stage between the translocation of the AAV genome from the endocytic to the nuclear compartment and the initiation of viral gene expression [15]. Lately, mutational analysis of amino acid residues involved in AAV2 capsid pore architecture indicate that conformational changes of the virion structure during infection lead the VP1 N termini to protrude through the capsid pores inducing the PLA2 enzymatic activity needed for successful infection [13]. At the level of virion formation, immunofluorescence data shows that the VP1 and VP2 proteins are found primarily in the nuclei of infected cells, whereas VP3 is nearly evenly distributed between the nucleus and the cytoplasm [16]. However, in the presence of VP1 and/or VP2, VP3 accumulates in the nucleus suggesting transport of the major capsid protein by association with the nuclear localization signal-bearing proteins VP1 and VP2 [17]. Immunofluorescence results suggest that capsid assembly is confined to the nucleoli of infected cells. The involvement of nucleolar chaperones in this process has been postulated [16]. Fully assembled AAV capsids enter the nucleoplasm in an AAV Rep-dependent manner. This redistribution of the structural proteins causes the co-localization of all ingredients necessary for infectious particle formation, i.e., capsids, Rep proteins and viral genomes. Indeed, the AAV DNA packaging process is though to take place in distinct regions of the nucleoplasm [16]. Selective AAV DNA encapsidation is presumably directed by protein-protein interactions between pre-formed empty capsids and complexes of Rep78 or Rep68 with the virus genome [18]. Next, the helicase domains of capsid-docked Rep52 and Rep40 proteins are proposed to act as molecular motors that unwind and transfer *de novo *synthesized single-stranded DNA into empty particles [19] through the pores located at the fivefold symmetry axes [13].

### Host cell infection

AAV2 virions utilize as primary attachment receptor heparan sulphate proteoglycans [20] while internalisation is aided by the co-receptors α_v_β_5 _integrin heterodimers [21], fibroblast growth factor receptor type 1 [22] and the hepatocyte growth factor receptor, c-Met [23]. The use of ubiquitous heparan sulphate proteoglycans as docking sites explains in part the well-known broad tropism of this virus that include, human, non-human primate, canine, murine and avian cell types. AAV5 and AAV4 also bind to charged carbohydrate moieties in the form of N- and O-linked sialic acids, respectively [24]. Expression profiling of AAV5 permissive and non-permissive cells with cDNA microarrays led to the identification of platelet-derived growth factor receptor as another cellular determinant involved in AAV5 infection [25].

The events and processes that regulate the trafficking of AAV particles into the nucleus are still not fully understood, however, some findings have been reported. For instance, infection experiments in HeLa cells expressing a dominant-negative form of dynamin significantly reduced AAV2 entry [26,27]. These results indicate that one route by which this virus can poke through the plasma membrane involves receptor-mediated endocytosis via the formation of clathrin-coated pits. In addition, lysomotropic agents and proton pump inhibitors greatly hamper AAV2 infection suggesting that internalised virions escape from endosomes and are released in the cytosol by a low pH-dependent process [27]. In addition, a powerful new imaging technique based on single-molecule labelling of discrete AAV particles enabled real-time monitoring of the trajectories of individual virions [28]. In these experiments, it was shown that each endosome carries a single AAV particle. Moreover, the abrogation of vectorial motion of virions in nocodazole-treated cells supported the involvement of microtubule assembly and motor proteins in active AAV intracellular transportation. Finally, it has been suggested that AAV particles due to their very small size can access the nucleus through the nuclear pore complex (NPC). However, recent research points to a nuclear entry process that is not dependent on NPC activity [29,30] whereas the issue of whether AAV capsids enter nuclei intact or remodelled seems to depend on the presence or absence, respectively, of co-infecting helper Ad particles [30].

### Lytic and lysogenic pathways

After entry into the host cell nucleus, AAV can follow either one of two distinct and interchangeable pathways of its life cycle: the lytic or the lysogenic. The former develops in cells infected with a helper virus such as Ad or herpes simplex virus (HSV) whereas the latter is established in host cells in the absence of a helper virus. When AAV infects a human cell alone, its gene expression program is auto-repressed and latency ensues by preferential integration of the virus genome into a region of roughly 2-kb on the long arm (19q13.3-qter) of human chromosome 19 [31,32] designated *AAVS1 *[33]. Recent research showed that this locus is in the vicinity of the muscle-specific genes *p85 *[34], *TNNT1 *and *TNNI3 *[35]. Furthermore, the *AAVS1 *sequence lies in a chromosomal region with characteristics of a transcription-competent environment [36]. Interestingly, an insulator within this locus was recently identified [37]. The targeted integration of the AAV genome, a phenomenon unique among all known eukaryotic viruses, enables the provirus DNA to be perpetuated through host cell division. Moreover, the level of specificity of this process of AAV biology (a single preintegration region within the entire human genome) makes its exploitation highly attractive for achieving the ultimate goal of safe and stable transgene expression [38].

Even if working models for the targeted DNA integration mechanism remain sketchy [39,40], the viral components needed for the site-specific integration reaction have been identified. They are composed in *cis *by the AAV ITRs and in *trans *by either one of the two largest Rep proteins (i.e., Rep78 or Rep68). Recently, another *cis*-acting sequence was shown to be necessary for high-level site-specific DNA integration [41,42]. This sequence overlaps with the highly regulated p5 promoter and, like the ITR sequence, harbours an RBE.

Detailed genetic analyses using an *AAVS1*-containing episome system demonstrated that a 33-bp sequence containing elements related to the RBE and to the trs is sufficient for targeted DNA integration. Their functional relevance was demonstrated by the absence of targeted DNA integration into mutated substrates [39]. In addition, the *AAVS1 *region behaves as an origin of replication in the presence of Rep proteins both *in vitro *[43] and *in vivo *[44]. Finally, the *AAVS1*-specific RBE and trs are separated by a spacer element whose sequence and length affects the efficiency of the site-specific DNA integration reaction [45]. The human genome has numerous Rep binding sites. However, database searches have revealed that an RBE at a proper distance from a trs sequence occurs only in the *AAVS1 *locus, which is consistent with the specificity of the integration reaction revealed through biological assays [46]. Moreover, *in vitro *studies showed that via their interaction with the RBE sequences present in the AAV ITRs and in the *AAVS1 *locus, Rep78 and Rep68 proteins could tether viral to cellular DNA [47]. Although, as mentioned above, the actual mechanism evolved by AAV to target its DNA to the *AAVS1 *locus is currently unknown, taken together these observations provide at the molecular level an explanation for the specificity of the reaction and the requirement for RBE-containing sequences in *cis *and either one of the two largest Rep proteins in *trans*. Remarkably, only recently a study emerged directly addressing the AAV DNA integration efficiency and the correlation between random versus targeted integration levels [48]. Using a tissue culture system, the authors showed by clonal analyses of target cells and Southern blot hybridisations that 50% of infected cells were stably transduced by AAV when a multiplicity of infection of 100 was used. Raising the dose of virus increased neither the frequency of infected cells nor the integration levels. Although multiplicities of infection of 100 and 10 both yielded approximately 80% infected cells, the frequency of stably transduced cells was below 5% when employing the lower dose. Virtually all integration events targeted the *AAVS1 *locus. Finally, for each multiplicity of infection, the frequency of *AAVS1 *site disruption without accompanying DNA insertion was higher than the frequency of site-specific integration by a factor of 2.

When a latently infected cell is super-infected with a helper virus, the AAV gene expression program is activated leading to the AAV Rep-mediated rescue (i.e., excision) of the provirus DNA from the host cell chromosome followed by replication and packaging of the viral genome. Finally, upon helper virus-induced cell lysis, the newly assembled virions are released. The induction of the lytic phase of the AAV life cycle from a stably integrated provirus can also occur in the absence of a helper virus, though with a lower efficiency, when the host cell is subjected to metabolic inhibitors and to DNA damaging agents such as UV irradiation or genotoxic compounds [49]. Moreover, in differentiated keratinocytes of an epithelial tissue culture system modelling skin, AAV2 was shown to initiate and proceed through a complete replicative cycle in the absence of helper viruses or genotoxic agents [50]. Taken together, these phenomena indicate that AAV is not defective in absolute terms.

## Adeno-associated virus vectorology

### General principle

**Figure 4:**
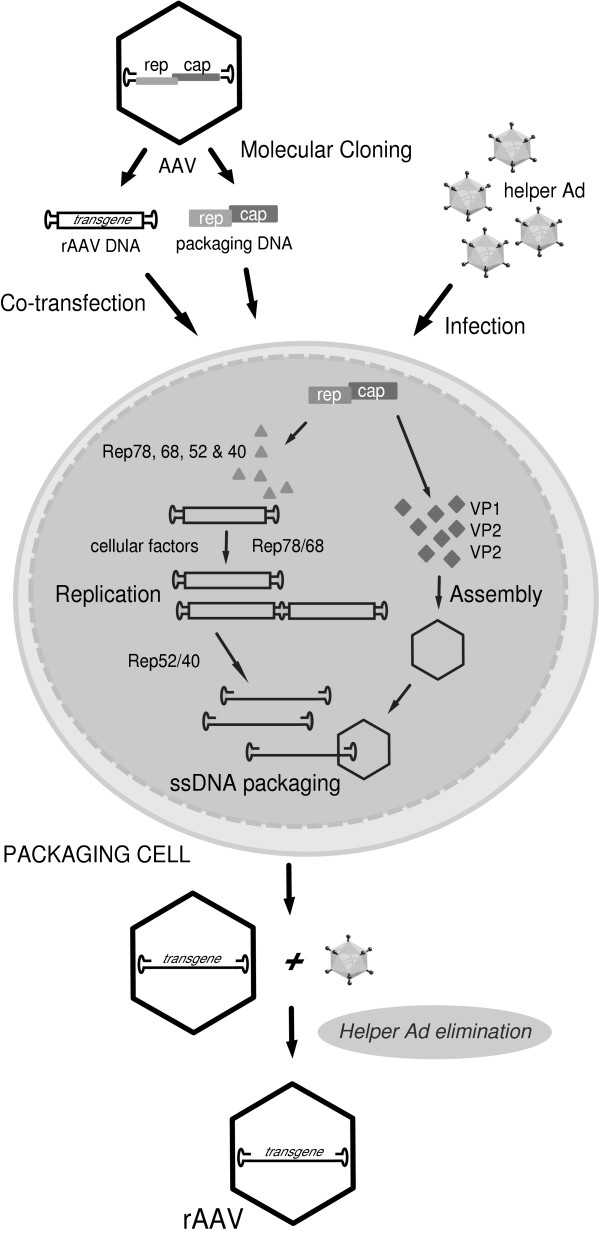
Overview of the initial recombinant AAV production system. The generation of the first infectious clones of AAV permitted functional dissection of the virus genome. This allowed the construction of plasmids encoding rAAV genomes in which the minimal complement of wild-type sequences necessary for genome replication and packaging (i.e., the AAV ITRs) frame a gene of interest (transgene) instead of the AAV *rep *and *cap *genes. When these constructs are transfected into packaging cells together with a *rep *and *cap *expression plasmid they lead to the production of rAAV particles. Helper activities required for the activation and support of the productive phase of the AAV life cycle were originally introduced by infection of the packaging cells with wild-type Ad as depicted. Current transfection-based production methods make use of recombinant DNA encoding the helper activities instead of Ad infection. Cellular DNA polymerase activities together with the Rep78 and Rep68 proteins lead to the accumulation of replicative intermediates both in the duplex monomer (DM) and duplex dimer (DD) forms. A fraction of this *de novo *synthesized DNA is incorporated in the single-stranded format into preformed empty capsids most likely through the catalytic activities of the Rep52 and Rep40 proteins. The resulting infectious rAAV virions are released from the producer cells together with helper Ad particles. Sequential heat treatment and buoyant density centrifugation allows the selective elimination of the helper virus from the final rAAV preparation.

Historically, most recombinant AAV (rAAV) vectors have been based on serotype 2 (AAV2) that constitutes the prototype of the genus [51,52]. Important to those pursuing the use of rAAV for gene therapy applications is the defectiveness of the parental virus and its presumed non-pathogenic nature. The realization that a molecularly cloned AAV genome could in Ad-infected cells recapitulate the lytic phase of the AAV life cycle and give rise to infectious virions enabled not only the detailed genetic analyses of the virus but provided, in addition, a substrate to generate rAAV particles [53]. The latter task was facilitated by the fact that the AAV ITRs contain all *cis*-acting elements involved in genome rescue, replication and packaging. Furthermore, since the AAV ITRs are segregated from the viral encoding regions, rAAV design can follow the whole-gene-removal or "gutless" vector rational of, for instance, retrovirus-based vectors in the sense that the *cis*-acting elements involved in genome amplification and packaging are in linkage with the heterologous sequences of interest, whereas the virus encoding sequences necessary for genome replication and virion assembly are provided in *trans *(Fig. 4). Typically, rAAV particles are generated by transfecting producer cells with a plasmid containing a cloned rAAV genome composed of foreign DNA flanked by the 145 nucleotide-long AAV ITRs and a construct expressing in *trans *the viral *rep *and *cap *genes. In the presence of Ad helper functions, the rAAV genome is subjected to the wild-type AAV lytic processes by being rescued from the plasmid backbone, replicated and packaged into pre-formed AAV capsids as single-stranded molecules.

### Production and purification strategies

The Ad helper functions were originally supplied by infection of rAAV producer cells with a wild-type Ad (Fig. 4). Subsequent elimination of the helper virus from rAAV stocks relied on the distinct physical properties of AAV and Ad virions. In particular, differences in thermostability and density between AAV and Ad particles allowed the specific elimination of helper Ad virions by heat-inactivation (i.e., half-hour at 56°C) and isopycnic cesium chloride density ultracentrifugation. The finding that Ad helper functions are provided by expression of *E1A*, *E1B*, *E2A*, *E4ORF6 *and *VA *RNAs, enabled subsequent Ad-free production of rAAV vector stocks by incorporating *VA *RNAs, *E2a *and *E4ORF6 *sequences into a plasmid and transfecting it together with the rAAV DNA plus *rep *and *cap *templates into Ad *E1A*- and *E1B*-expressing cells [54-56]. During the testing of new packaging plamids for rAAV production it was also found that reduction of the expression levels of the two largest AAV Rep proteins leads to an increase in vector yields [56,57]. Although these methods improve rAAV production and avoid the need for Ad infection, they are difficult to scale up due to their dependence on DNA transfection. The development of up-scalable transfection-independent methods for rAAV production have been fiercely pursued by the requirement for large amounts of highly purified vector particles to perform experiments in large animal models and human clinical trials. One of these transfection-independent production strategies involves the generation of packaging cell lines having the AAV *rep *and *cap *genes stably integrated in their genomes. The establishment of effective, high-titer producer cell lines has proven difficult mainly due to the inhibitory effects of Rep proteins on cell growth [58] and the accumulation of low amounts of AAV gene products relative to a wild-type virus infection. Nonetheless, improvements in the control of *rep *expression through the development of stringent inducible gene expression systems can overcome the former hurdle [59] whereas *in situ *amplification of integrated *rep *and *cap *templates helps to minimize the latter problem [60,61]. Another transfection-independent approach to produce rAAV involves the delivery of the viral genes together with the rAAV DNA and the helper functions via infection of produced cells with recombinant viruses based on Ad [60], HSV [62] or baculovirus [63]. In parallel to new rAAV production platforms, insights into AAV biology are also leading to significant improvements in the quality and purity of vectors based on AAV2 as well as on those based on other serotypes. Specifically, knowledge on AAV receptor usage has permitted the implementation of up-scalable affinity column chromatography purification schemes [64,65]. In addition, a more broadly applicable column chromatography procedure, based on the ion-exchange principle, has recently been developed for the purification of rAAV2, rAAV4 and rAAV5 particles [66].

### Tropism modification

An increasingly important area in the development of AAV as a vector concerns the engineering of altered cell tropisms to narrow or broaden rAAV-mediated gene delivery and to increase its efficiency in tissues refractory to AAV2 infection. Cells can be poorly transduced by prototype rAAV2 not only because of low receptor content but also owing to impaired intracellular virion trafficking and uncoating [67,68] or single-to-double strand genome conversion [69-71]. Thus, considering that these processes depend either directly or indirectly on capsid conformation, cell targeting strategies determine not only the cell type(s) with which the vector interacts but also critically affect the efficiency of the whole gene transfer process.

Several of these approaches rely on the modification by chemical, immunological or genetic means of the AAV2 capsid structure endowing it with ligands that interact with specific cell surface molecules [72]. The fact that the atomic structure of AAV2 has recently been determined [2] provides a significant boon to those pursuing the rational design of targeted AAV vectors. Another route to alter rAAV tropism exploits the natural capsid diversity of newly isolated serotypes by packaging rAAV2 genomes into capsids derived from other human or non-human AAV isolates [73]. To this end, up until now, most researches employ hybrid *trans*-complementing constructs that encode *rep *from AAV2 whereas *cap *is derived from the serotype displaying the cell tropism of choice. This pseudotyping approach may also be beneficial in evading neutralizing antibodies to capsid components in individuals seropositive for AAV2 or in those in need of vector readministration. Finally, experiments published recently using rAAV2 genomes pseudotyped with coats from AAV6 [74] and AAV8 [75] revealed stunning gene transfer efficiencies when these vectors were administered alone at high doses or in combination with a blood vessel permeating agent. The authors could demonstrate transduction of the entire murine striated muscle system (e.g., diaphragm, heart and skeletal muscles) and of virtually 100% of the hepatocytes after a single intravenous injection. These body-wide transduction efficiencies raise both great perspectives as well as caution since they open new therapeutic avenues for diseases that require widespread gene delivery (e.g., muscular dystrophies) while, simultaneously, beg for stringent tissue-specific transcriptional control to minimize potential deleterious effects due to transgene expression in non-target tissues. Moreover, assuming similar avidity of these serotypes for human tissues, translation of these protocols from mice to patients will require vastly greater amounts of vector particles.

### Mechanisms of vector DNA persistence

Knowledge on the mechanisms at play following rAAV transduction is building steadily over recent years mainly because of its direct relevance to the application of rAAV in therapeutic gene transfer. DNA vectored through rAAV can persist long-term in organs such as in the liver and the striated muscles of mice and dogs. Most importantly, data showing prolonged and stable expression of an increasing variety of transgenes in numerous animal models without notable toxicity is accumulating [76]. It are in fact these attributes of rAAV-based gene transfer that turns it into one of the most promising methods for somatic gene therapy providing a rational for the entry of these vectors into the clinical trial arena. However, at the outset it is important to refer that this stability does not arise due to foreign DNA insertion into the parental virus pre-integration site since the absence of *rep *gene products prevents DNA targeting to the *AAVS1 *locus. Moreover, because rAAV vectors lack viral genes altogether, the molecular fate of the DNA once in the nucleus is dependent on host cell activities (though a role for the virion capsomers cannot be ruled out). These cellular activities, that only recently have started to be identified, depend on the type as well as on the physiological status of the target cell. Finally, it is also of note that the single-stranded nature of AAV genomes implies that, before transgene expression can occur, the incoming rAAV DNA needs to be converted into a transcriptionally functional double-stranded template.

A recent study indicates that free (i.e., unpackaged) single-stranded rAAV genomes have a very transient presence in the target cell [67] either because the majority is recognized by host enzymes as damaged DNA and degraded or because, under certain conditions, single-to-double strand conversion occurs readily following uncoating. There are two pathways by which rAAV DNA can be converted from the single- to the double-stranded form each of them with its own set of supporting experimental data. One possible route develops through *de novo *second-strand DNA synthesis from the hairpin at the 3' end of the genome (Fig. 2). Initial studies revealed that this step could be greatly enhanced by Ad *E4ORF6 *expression, UV irradiation or treatment of target cells with genotoxic chemicals [69,70]. Furthermore, a direct correlation between double-stranded template accumulation and gene expression was found. More recently, the phosphorylation status of a cellular protein named FKBP52 was shown to modulate the convertion of single-stranded rAAV DNA into double-stranded molecules both in tissue culture [77] and in murine hepatocytes [78]. FKBP52 phosphorylation by the epidermal growth factor receptor protein tyrosine kinase enables the molecule to bind the single-stranded AAV ITR D-sequence (Fig. 2). This binding activity correlates strongly with second-strand DNA synthesis inhibition. Conversely, in its dephosphorylated state, due to T-cell protein tyrosine phosphatase activity, FKBP52 does not bind vector genomes allowing synthesis of the complementary strand to occur with a subsequent increase in transgene expression levels.

As said before, single-stranded AAV genomes with sense (plus) and anti-sense (minus) orientations are packaged equally well. Therefore, another possible route involved in the generation of double-stranded DNA forms in target cells comprises the annealing of single-stranded molecules with opposing polarities. Evidence for the existence of this DNA synthesis-independent pathway came from experiments using rAAV genomes that were site-specifically methylated [71]. In these experiments restriction enzymes were used as probes to evaluate whether modified rAAV genomes extracted from murine livers were fully methylated (representing annealing products) or hemimethylated (corresponding to second-strand synthesis products). Thus, seemingly, a contention exits between advocates of DNA synthesis dependent and independent models. It is clear, however, that these two pathways are not necessarily mutually exclusive. In fact, recent experiments in cells under normal physiological conditions indicate that each of these pathways can contribute to the generation of transcriptionally active rAAV genomes [67]. For the latter experiments the authors resurrected a technique deployed to directly demonstrate that AAV is a single-stranded virus [8]. Exploiting the differential thymidine content of complementary polynucleotide chains they used incorporation of the thymidine analogue bromodeoxyuridine (BrdU) to physically separate plus- from minus-strand containing rAAV particles following buoyant density centrifugation. Infection of indicator cells with each vector type led to reporter gene expression signifying the involvement of second-strand DNA synthesis and precluding an absolute requirement for plus and minus strand annealing. However, co-infection with both vector types originated higher numbers of cells expressing the reporter gene indicating that strand annealing contributes to the accumulation of double-stranded genomes [67].

Subsequently, duplex rAAV genomes can, throught intra- or intermolecular recombination at the ITRs, originate circular forms or linear concatemers, respectively [71,79]. The circular episomes can also evolve into high-molecular-weight concatamers in a time-dependent manner [79]. The balance between linear versus circular forms is, at least in part, regulated by a complex containing DNA-dependent protein kinase (DNA-PK) [80]. This complex plays a vital role in the repair of double-stranded chromosomal breaks and in V(D)J recombination by non-homologous end-joining (NHEJ). The absence of the catalytic subunit of DNA-PK (DNA-PKcs) in severe combined immunodeficient (SCID) mice (DNA-PKcs-negative) allowed Song and colleagues to demonstrate its involvement in circular rAAV episome formation in skeletal muscle [80]. Subsequent studies in liver and skeletal muscle of SCID and normal (DNA-PKcs-positive) mice have extended the observation that DNA-PK enhances the formation of rAAV circular episomes over linear forms [81,82]. It has been postulated that free double-stranded rAAV DNA ends are substrates for the cellular double-stranded break repair machinery responsible for free-ended DNA removal through NHEJ ligation [80]. Notwithstanding their diverse topology and unit numbers, all these extrachromosomal DNA forms are transcription-competent templates. Furthermore, they are thought to be responsible for the stable maintenance of transgene expression both in skeletal muscles [79] and in the lungs [83]. In the liver it has been shown that, in addition to the aforesaid episomal forms, *circa *10% of the double-stranded rAAV genomes can be found inserted in the chromosomal DNA [84].

Backed by the complete mouse genome sequence, researchers could establish that a significant proportion of rAAV DNA integration events occur in regions that are transcriptionally active in murine hepatocytes [85]. In some instances, sequence micro-homologies and unrelated nucleotides are found at the truncated ITR-chromosomal DNA junctions. Moreover, rAAV DNA insertion is consistently associated with host chromosomal deletions. These characteristics resemble the "fingerprints" following double-stranded DNA break repair through NHEJ recombination. Thus, taken together, these results point to the involvement of NHEJ in rAAV DNA integration in addition to its putative role in the removal of free rAAV DNA ends, as previously discussed. This interpretation is further supported by previous and newly acquired data. For instance, earlier tissue culture studies revealed a direct correlation between genomic instability due to DNA-damaging agents or genetic defects and stable transduction by rAAV [86,87]. Other results showed that proteins belonging to the NHEJ complex bind to linear rAAV DNA [88]. More recently, a genetic approach permitted the deliberate induction of double-stranded chromosomal breaks within a predefined site [89]. The experimental set up consisted of retrovirus vector-mediated expression of the I-*Sce*I endonuclease in cells engineered with this enzyme's 18-bp recognition sequence. Following transduction of these cells with rAAV, the authors could demonstrate insertion of foreign DNA into I-*Sce*I-induced double-stranded breaks. Characterization of vector-chromosome junctions revealed the telltale features observed after rAAV DNA integration into chromosomal breaks arising spontaneously at random sites. It is thus possible to speculate that rAAV proviral DNA is just another by-product of the mechanism the cell uses to eliminate free-ended substrates reminiscent of damaged DNA or invading nucleic acids (e.g., linear retroviral cDNA). As corollary, compared to the integrase-dependent retroviral genome integration, rAAV DNA insertion is a passive process that relies instead on pre-existent chromosomal breaks and host cell enzymes.

Chromosomal DNA integration with current vectors is a double-edged sword. On the one hand it provides a basis for permanent genetic correction while, on the other hand, raises safety issues related to insertional gene-inactivation and proto-oncogene deregulation. It is thus highly relevant for the clinical deployment of rAAV that these vectors do not create but instead insert into existing chromosomal breaks. The latter can be substrates for inaccurate NHEJ-mediated repair regardless of the presence of rAAV genomes. Therefore, concerns about insertional oncogenesis might be less for rAAV- than for retroviral vector-mediated gene transfer. Additionally, in contrast to retroviral vectors, rAAV vectors do not display "outward" promoter activity. Despite this, it is still conceivable that rAAV DNA insertion can lead to hazardous alteration of neighbouring gene(s) expression via vector-encoded regulatory sequences (e.g., enhancers). Thus, preventive measures such as judicious choice of transcriptional elements and use of insulators may turn out to be desirable or even indispensable in target tissues in which rAAV DNA is known to integrate at appreciable levels. Adding to the challenge these genetic elements have to be small enough to leave space needed to accommodate the gene of interest.

### Emerging technologies

**Figure 5:**
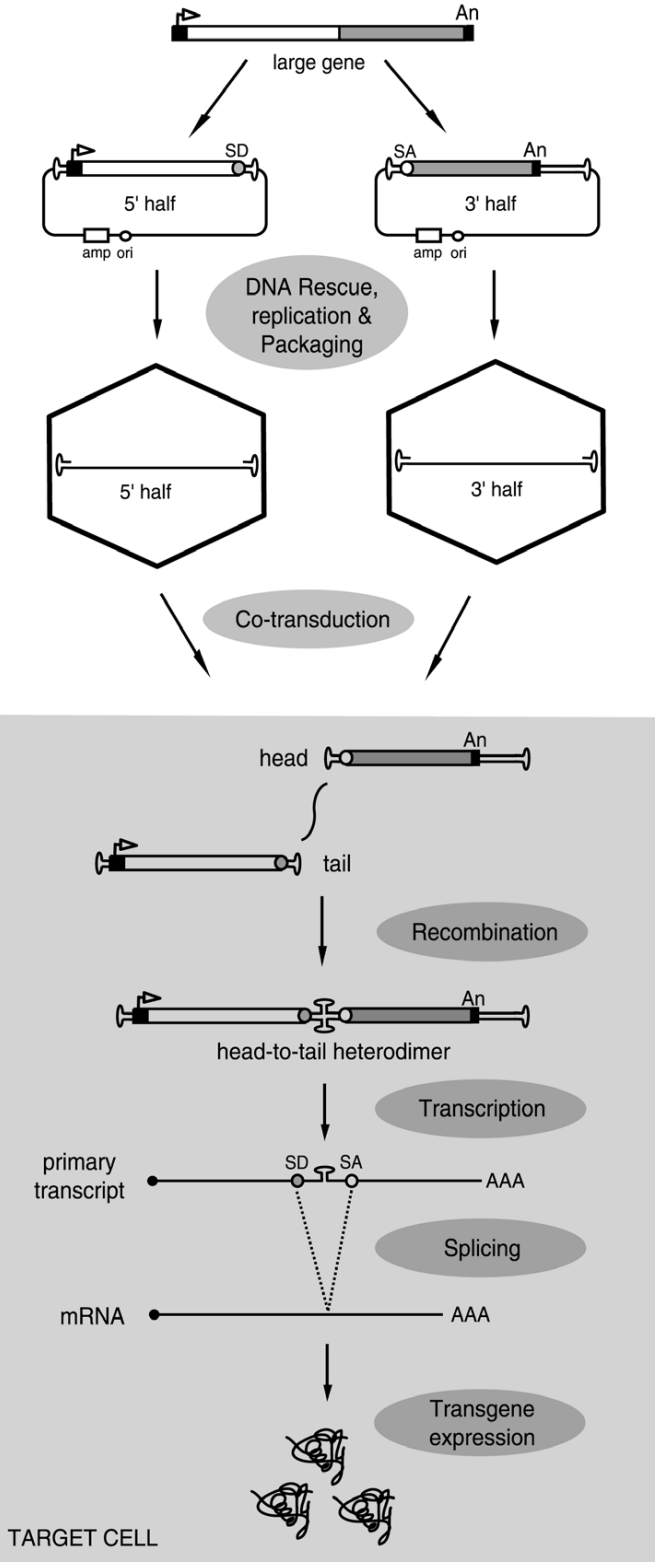
Diagram of the recombinant AAV split gene principle. An expression unit corresponding to a large gene is roughly divided in two halves. One of them consists of a promoter (solid box with arrowhead), the 5' half of the gene (open box) and a splice donor site (SD) while the other encodes a splice acceptor sequence (SA), the 3' portion of the gene (shaded box) and a polyadenylation signal (solid box). These fragments are independently cloned between two AAV ITRs. Vector stocks are then generated from the resulting shuttle plasmids and are used to co-transduce target cells. Head-to-tail heterodimerization via intermolecular recombination between the two vector DNA molecules restores the full-length expression unit and results in the synthesis of the desired protein after the splicing of the intervening AAV ITR sequences from the primary transcript.

The small packaging capacity of AAV particles (about 4.7 kb) [90] is considered one of the main limitations of rAAV vectors since it excludes therapeutically important coding sequences (e.g., dystrophin cDNA) and potent regulatory elements (e.g., albumin promoter). As discussed above, incoming linear rAAV genomes can form concatamers in target cells through intermolecular recombination at their free ends. This phenomenon has been successfully exploited to assemble in target cells large genetic messages through the joining of two independently transduced rAAV genomes each of which encompassing a portion of a large transcriptional unit. mRNA molecules encoding a functional protein are generated from the rAAV DNA head-to-tail heterodimers by splicing out the AAV ITR sequences from the primary transcripts (Fig. 5) [91]. Although this split gene strategy allows expression of almost double-sized transgenes after rAAV-mediated gene delivery, its efficiency is consistently lower than that observed with a single control vector encoding the full-length transgene. Both vectors have to transduce the same cell and only heteroconcatamers with a head-to-tail organization will give rise to a functional full-length gene product. In addition, there are risks associated with the integration into host chromosomes of vectors encoding exclusively regulatory elements or truncated gene products. New work, however, suggests that some of these limitations and concerns can, at least partially, be addressed [92,93].

**Figure 6:**
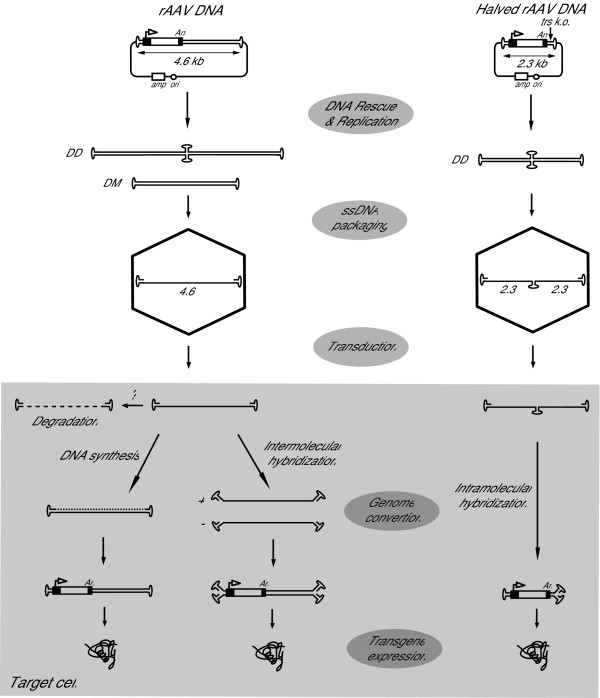
Diagram of the generation and transduction of a self-complementary AAV vector as compared to that of a conventional recombinant AAV. Left panel: According to the AAV DNA replication scheme, full-length rAAV genomes of both polarities are generated from duplex monomeric (DM) and duplex dimeric (DD) replicative intermediates and individually packaged in AAV capsids. In the nucleus of transduced cells the single-stranded genomes can either be a target for degradation or be converted into transcriptionally active double-stranded templates. The single-to-double strand DNA conversion depends on complementary chain synthesis or on the recruitment of a complementary genome (i.e., intermolecular hybridization). Right panel: According to the same replication model, a rAAV genome with roughly half the size of the wild-type AAV DNA and with one trs mutated, generates DD replicative intermediates with an inverted repeat configuration containing wild-type ITRs at the extremities and mutated ITRs at the axis of symmetry. Single-stranded molecules derived from these DNA structures are packaged in AAV capsids. After uncoating in the target cell nucleus, these molecules can readily fold into double-stranded templates through intramolecular base pairing due to their self-complementary nature (i.e., intramolecular hybridization).

Another development in rAAV design is the so-called self-complementary AAV vectors (scAAV) [94]. The scAAV approach builds on the ability of AAV to package replicons with half the size of the wild-type DNA in the form of single-stranded dimeric genomes with an inverted repeat configuration [95]. In the target cell, these self-complementary molecules can readily fold back into double-stranded forms without the need for *de novo *DNA synthesis or for the annealing of sense and antisense strands (Fig. 6). Ultimately, regardless of the mechanism(s) at play, scAAV lead to enhanced formation of transcription-competent double-stranded genomes thus improving the expression kinetics and yields of vector-encoded products. This scAAV method was subsequently perfected by mutagenesis of one of the two trs sequences to force the generation of dimeric over monomeric replicative forms (Fig. 6) [96]. The main disadvantage of this approach is the need to limit the size of the transgenes that can be delivered to approximately half the length of the already small AAV genome. It is conceivable that this drawback can be tackled by coupling scAAV with heterodimerization strategies. Alternatively, long double-stranded rAAV genomes can be transferred into target cells via capsids of larger viruses such as Ad [97-100], baculovirus [101] or HSV [102]. In some of these hybrid viral vector systems, integration of the rAAV DNA into the *AAVS1 *locus on human chromosome 19 was accomplished by transient expression of AAV Rep activities in the target cells [38]. Targeted DNA integration is advantageous since it dispels the insertional oncogenesis concerns discussed above.

Site-specific or targeted DNA integration can also be achieved through homologous recombination (HR) between a transduced DNA fragment and an endogenous gene in the target cell genome. The ability to introduce precise genetic modifications in germ cells of mice combined with powerful selection markers has revolutionized mammalian genetics [103]. The same principle can be applied to achieve correction of defective genes in somatic human cells. In fact, targeted gene correction is conceptually an attractive alternative to gene addition since there is no strict need to transduce the entire gene and associated regulatory elements but only a fraction of the targeted gene sequence. In addition, the corrected gene remains in its chromosomal context thus being subject to the proper regulatory circuitry. However, gene targeting strategies are currently not practical mostly due to the inefficiency of HR after foreign DNA delivery (typical frequencies lie below 10^-6^). It has been demonstrated that rAAV can be tailored to introduce precise nucleotide alterations in the genome of human cells at frequencies approaching 1% when multiplicities of infection in the order of 10^5 ^to 10^6 ^infectious genomes per cell are used [104]. In these experiments, it was observed that for each targeted integration event 10 non-targeted DNA insertions occurred and that, in comparison with other methods, the HR process was less dependent on the extent of homology. More recently, this technology was successfully used in human mesenchymal stem cells to disrupt via HR a mutant *COL1A1 *allele coding for a dominant-negative type of collagen causing osteogenesis imperfecta [105].

### Clinical trials

Data on safe and long-lasting rAAV-mediated transgene expression in organs of animal models of human disease such as lung, liver, central nervous system and eye, together with improvements in vector production and purification methods provided the rational for initiating clinical studies with rAAV vectors. Currently, these clinical trials are either in phase I or in phase II. The former studies aim at determining safety and often also maximum tolerable dose of the therapeutic agent, while the latter entail the assessment of its efficacy and have higher statistical significance to detect potential side effects. Ailments being targeted include Parkinson's disease, Canavan's disease, α1-antitrypsin deficiency, cystic fibrosis (cystic fibrosis transmembrane conductance regulator [CFTR] deficiency) and hemophilia B (blood clotting factor IX [FIX] deficiency). Cystic fibrosis and hemophilia B are two examples of which more information is available. In fact, more than one decade ago, cystic fibrosis patients were the first human individuals subjected to rAAV administration [106].

Cystic fibrosis is the most common autosomal recessive disorder among Caucasians. The *CFTR *gene encodes a chloride channel that is essential for the transport of chloride ions across the membranes of epithelial cells of the lungs, gastrointestinal tract and sweat glands. The CFTR aids in the physiological transport of other ions and water. The pathophysiology of cystic fibrosis in the lung is not settled [107]. However, it seems uncontroversial that in the absence of functional CFTR, mucus of high viscosity and abnormal ionic content covers the airway epithelium leading to the accumulation of infectious agents. Chronic inflammation results in lung tissue damage and loss of respiratory function. Early death ensues.

As said before, all clinical trials are based on preclinical data retrieved from experiments in animal models. Unfortunately, *CFTR *knockout mice display primarily intestinal defects as opposed to the lung deterioration typical of the human condition. Accordingly, New Zealand white rabbits [108] and rhesus monkeys [109] constituted the major preclinical models for rAAV-mediated *CFTR *cDNA transfer. Overall, these studies showed that transduction with AAV2-based vectors led to prolonged and dose-dependent *CFTR *cDNA expression in the respiratory tract after various modes of administration (e.g., direct bronchoscopic instillation and aerosol delivery). Importantly, no overt signs of vector-associated inflammation or toxicity were observed. Equally important, vector DNA was not detected in the gonads of any of the experimental animals tested, indicating that the risk of inadvertent germline transmission is very low. Initial clinical results showed rAAV2-mediated *CFTR *delivery to be well tolerated by human patients as well. It is also known from phase I dose-escalation studies that the aerosol method permits the delivery of vector DNA throughout the lung in a dose-dependent manner. Although vector sequences persisted for up to 90 days at the highest dose, vector-specific transcripts could not be detected in the samples tested [110]. A follow up placebo-controlled phase II study incorporated into its design repeated administration of aerosolized vector particles. In addition to safety monitoring, this trial included the evaluation of proinflammatory cytokine interleukine-8 (IL-8) levels and pulmonary function. The treatment was well tolerated and, at days 30 and 14, vector-treated patients showed evidence of improved lung function and reduced IL-8 concentrations in the sputum, respectively, when compared to placebo-treated individuals [111]. On the basis of these promising results new and expanded phase II clinical trials are currently underway.

In contrast to the mouse model of cystic fibrosis, *FIX *knockout mice and naturally occurring *FIX*-defective canines with missense and null mutations accurately mimic hemophilia B in humans. In addition, this X-linked coagulopathy has other features that turn it into an attractive target for gene transfer approaches. Firstly, the limited size of the *FIX *cDNA (i.e., 2.8 kb) allows the testing of a large variety of gene delivery systems including those with a small packaging capacity. Secondly, regulation of *FIX *expression is not needed because the encoded product has a broad therapeutic index and, importantly, concentrations above 1% of the physiological level start to be beneficial (i.e., < 1, 1 to 5, and > 5% correspond to severe, moderate and mild disease, respectively). Finally, although the liver is the normal site of FIX production, synthesis and secretion of a biologically active form of this protein can also be achieved from ectopic, easily accessible, tissues such as skeletal muscle. Indeed, sustained dose-dependent therapeutic levels of canine *FIX *expression were attained in hemophilic dogs after both portal vein [112] and intramuscular [113] injections of rAAV2 particles. Partial phenotypic correction could be unambiguously established in these studies by measurement of hemostatic parameters such as the whole blood clotting time (WBCT) and the activated partial thromboplastin time (aPTT) lending support for the testing of rAAV2 in patients. In 1999, a dose-escalation phase I trial consisting of three dose cohorts (i.e., 2.0 × 10^11^, 6.0 × 10^11^, and 1.8 × 10^12 ^vector genomes per kilogram of body weight) with three patients each was initiated. The readily accessible *vastus lateralis *muscle was chosen as target tissue for safety reasons. Results from these first parenteral administrations of rAAV in human subjects showed safe transfer of *FIX *without evidence for the formation of inhibitory antibodies to FIX and for the presence of vector sequences in semen. Gene transfer was detected by PCR and Southern blot analyses, whereas immunohistochemical staining of muscle biopsies revealed sustained transgene expression distributed mainly in slow twitch fibers [114]. However, this trial also showed that the doses tested were too low to bring about FIX plasma concentrations decisively above 1% of the normal value. It became apparent that therapeutic doses required numerous injections with more particles being administered per site. Several issues, however, blocked this approach. Firstly, the number of injections needed rendered the procedure impractical. Secondly, it was considered that saturation of the AAV2 receptors and of the capacity of myocytes to secrete FIX with the correct posttranslational modifications [115] would curtail the effect of using very high particle concentrations. Finally, and most importantly, a correlation was observed between injection of very high dosages of rAAV2 into muscle and the development of FIX neutralizing antibodies [113].

The next phase I trial targeted the liver of individuals with missense mutations by systemic administration of FIX-encoding rAAV2. Unfortunately, this trial has been halted. Low vector doses were well tolerated but did not induce FIX levels above baseline, whereas high vector doses achieved only transient *FIX *expression and induced hepatotoxicity and immune responses against the vector and the transgene product [116]. Hopefully, new developments in rAAV technologies such as, vectors endowed with regulatory elements for high-level tissue-specific expression and higher liver and/or muscle tissue avidities will increase the therapeutic potency of rAAV-mediated *FIX *transfer in humans. Towards this goal, intraportal administration of an AAV8-based vector directing the synthesis of canine FIX through a liver-specific promoter achieved stable curative levels of the protein in naïve and in AAV2-preimmunized hemophilia B dogs (i.e., up to 26% and 16% of normal levels, respectively) [117]. The results obtained in AAV2-pretreated dogs are particularly significant if one considers that a significant proportion of humans have high AAV2 neutralizing antibody titers [118].

## Conclusion

Important strides have recently been made in the optimisation of rAAV technology at the levels of production and performance. Insights from AAV biology have been instrumental in this process and are expected to continue to be the main catalyst behind the further development and efficacious deployment of rAAV. Most of the features initially identified in AAV as being highly desirable in a therapeutic gene carrier such as the seemingly nonpathogenic nature of the wild-type virus and its ability to infect, non-dividing, terminally differentiated cells remain valid and contribute to put rAAV at the forefront of all vector systems that aim at safe and sustained transgene expression *in vivo*. A notable exception of an AAV attribute not retained by rAAV concerns the loss of *AAVS1*-targeted DNA integration.

The number of promising reports documenting rAAV-mediated stable transgene expression in immunocompetent recipients is steadily increasing. However, the vast majority of these results have been obtained in inbred rodent models with relatively little genetic diversity. There are several indications (e.g., from research on rAAV-mediated *FIX *transfer) that the results obtained in mice cannot predict the outcome of experiments carried out in patients. This underscores the need not only for continuous improvement of the vectors themselves but also for deepening the knowledge about vector-host interactions outside the realm of rodent models. The ultimate goal of this research is to accomplish unequivocal clinical benefit by the identification of limitations and corresponding solutions to each particular disease-transgene-vector trilogy.

## Competing interests

The author(s) declare that they have no competing interests.
